# Apoptosis-associated microRNAs are modulated in mouse, rat and human neural differentiation

**DOI:** 10.1186/1471-2164-11-514

**Published:** 2010-09-24

**Authors:** Márcia M Aranha, Daniela M Santos, Joana M Xavier, Walter C Low, Clifford J Steer, Susana Solá, Cecília MP Rodrigues

**Affiliations:** 1 Research Institute for Medicines and Pharmaceutical Sciences, Faculty of Pharmacy, University of Lisbon, Lisbon 1649-003, Portugal; 2 Department of Neurosurgery, University of Minnesota Medical School, Minneapolis, MN 55455, USA; 3 Stem Cell Institute, University of Minnesota Medical School, Minneapolis, MN 55455, USA; 4 Department of Medicine, University of Minnesota Medical School, Minneapolis, MN 55455, USA; 5 Department of Genetics, Cell Biology, and Development, University of Minnesota Medical School, Minneapolis, MN 55455, USA

## Abstract

**Background:**

MicroRNAs (miRs or miRNAs) regulate several biological processes in the cell. However, evidence for miRNAs that control the differentiation program of specific neural cell types has been elusive. Recently, we have shown that apoptosis-associated factors, such as p53 and caspases participate in the differentiation process of mouse neural stem (NS) cells. To identify apoptosis-associated miRNAs that might play a role in neuronal development, we performed global miRNA expression profiling experiments in NS cells. Next, we characterized the expression of proapoptotic miRNAs, including miR-16, let-7a and miR-34a in distinct models of neural differentiation, including mouse embryonic stem cells, PC12 and NT2N cells. In addition, the expression of antiapoptotic miR-19a and 20a was also evaluated.

**Results:**

The expression of miR-16, let-7a and miR-34a was consistently upregulated in neural differentiation models. In contrast, expression of miR-19a and miR-20a was downregulated in mouse NS cell differentiation. Importantly, differential expression of specific apoptosis-related miRNAs was not associated with increased cell death. Overexpression of miR-34a increased the proportion of postmitotic neurons of mouse NS cells.

**Conclusions:**

In conclusion, the identification of miR-16, let-7a and miR-34a, whose expression patterns are conserved in mouse, rat and human neural differentiation, implicates these specific miRNAs in mammalian neuronal development. The results provide new insights into the regulation of neuronal differentiation by apoptosis-associated miRNAs.

## Background

Adult neural stem (NS) cells and embryonic stem (ES) cells are capable of differentiating into multiple cell types of the adult nervous system [1,2]. This unique property promises future medical applications, ranging from regenerative medicine to drug screening. However, the therapeutic use of NS cells and ES cells will require the identification of cellular conditions that promote the efficient commitment of these progenitors to particular cell phenotypes.

Much progress has been made toward the elucidation of specific differentiation signaling pathways. Surprisingly, recent evidence suggests a functional role for apoptosis-associated proteins such as p53 [3,4], caspases [5] and Bcl-2 [6] in differentiation and development. In addition, the recent discovery of microRNAs (miRs or miRNAs) introduced a novel type of regulatory control of gene expression during developmental processes [7,8]. miRNAs are a class of endogenous noncoding, highly conserved RNAs of ~ 22 nucleotides in length [9]. They regulate mRNA expression either by translation repression or cleavage of targeted mRNA [8]. In the past few years, our understanding of miRNAs has expanded, and a role for apoptosis-associated miRNAs in cell differentiation is emerging.

Let-7a, a member of the let-7 family, is associated with apoptosis by directly targeting caspase-3 [10]. Let-7 was first identified in *Caenorhabditis elegans *and reported to control the timing of fate specification during larval development [11]. In addition, let-7a was also implicated in neuronal differentiation [12,13]. However, the mechanism by which let-7a regulates cell differentiation is unknown.

Members of the miR-34 family are direct p53 targets, and their upregulation induces apoptosis and cell cycle arrest [14-19]. In this regard, miR-34a has been shown to regulate genes involved in cell cycle control and apoptosis, including cyclin-dependent kinase 4 (CDK4), CDK6, cyclin D1, E2F3 and SIRT [17,20,21]. A role for miR-34a in megakaryocytic differentiation was recently reported, where the miRNA regulates the expression of MYB, and CDK4 and CDK6, thus promoting cell cycle arrest [22]. Furthermore, miR-34a is involved in dendritic cell differentiation [23], and is required for proper differentiation of mouse ES cells by targeting Sirt1 [24].

miR-16 is implicated in induction of apoptosis by targeting Bcl-2 [25], and is involved in cell cycle regulation by targeting CDK6, cell division cycle protein 27 (CDC27), the caspase recruitment domain-containing protein 10 (CARD10), cyclin D1 and cyclin E [26-28]. Nevertheless, an involvement of miR-16 in cell differentiation is virtually unknown.

To gain further insight into the role of apoptosis-associated factors during cell differentiation, we monitored specific apoptosis-associated miRNAs during NS cell differentiation. Our results showed that the differential expression of miR-16, let-7a and miR-34a during mouse NS cell differentiation was not associated with cell death. In addition, upregulation of these miRNAs observed in distinct models of neural differentiation strongly suggests that apoptosis-associated miRNAs may play a role in differentiation.

## Methods

### Cell Lines

#### Mouse NS Cells

Mouse NS cells containing a constitutively expressed marker for green fluorescent protein (GFP) were used to investigate the process of neural differentiation. Primary cells were obtained from central nervous system tissue of embryonic mice and cultured primarily as previously described [29-31]. Mouse NS cells were maintained as neurospheres in undifferentiation medium, serum-free, 1:1 mix of DMEM/F12 (Invitrogen Corp., Grand Island, NY) with 1 × N-2 supplement (Invitrogen Corp.), 20 ng/ml epidermal growth factor (EGF) (R & D Systems Inc., Minneapolis, MN), 20 ng/ml basic fibroblast growth factor (bFGF) (PeproTech EC, London, UK), and 1% penicillin-streptomycin (Invitrogen Corp.), at 37°C in humidified atmosphere of 5% CO_2_. Subculture occurred at day 7 with mechanical dissociation of neurospheres. The differentiation of mouse NS cells *in vitro *was induced by culturing dissociated cells in differentiation medium containing DMEM/F12 with 1 × N-2 supplement, 100 ng/ml bFGF, 10% fetal bovine serum (FBS) (Invitrogen Corp.), 500 nM all-*trans *retinoic acid (Sigma Chemical Co., St. Louis, MO), 50 μM taurine (Sigma Chemical Co.), 10 ng/ml transforming growth factor-β2 (TGF-β2) (R & D Systems Inc.) and 1% penicillin-streptomycin in tissue culture plates pre-coated with poly-D-lysine (Sigma Chemical Co.). The culture medium was changed every 3 days. Differentiated cells at 5 × 10^4 ^cells/ml were fixed at different time points, and processed for immunostaining and evaluation of differentiation. Cultures at 5 × 10^5 ^cells/ml were processed for western blot and PCR analysis.

In parallel experiments, cell death was prevented by adding tauroursodeoxycholic acid (TUDCA; Sigma Chemical Co.) to the culture medium. Briefly, cells with 3 days of differentiation were treated with 50 μM of TUDCA for 72 hours. At 6 days, cells were collected and processed for evaluation of apoptosis and miRNA expression.

miR-34a expression was modulated using 100 nM of precursor control and pre-miR-34a (Applied Biosystems, Foster City, CA). Mouse NS cells (2.5 × 10^5 ^cells/ml) were transfected at 3 or 5 days of differentiation using Lipofectamine 2000 (Invitrogen Corp) following manufacturer's instructions.

#### Mouse ES Cells

The ES cell line 46C (Sox1-GFP) [32] was grown and maintained as previously reported [33] and induced to differentiate using a monolayer protocol. Specifically, ES cells were plated in serum-free medium ESGRO Complete Clonal Grade medium (Millipor Inc.) at high density. After 24 hours, ES cells were gently dissociated and plated onto 0.1% (v/v) gelatin-coated tissue culture plastic at 1 × 10^4 ^cells/cm^2 ^in RHB-A media (StemCell Science Inc.). For replating on day 4, cells were dissociated and plated at 2 × 10^4 ^cells/cm^2 ^in laminin-coated tissue culture plastic, using RHB-A medium supplemented with 5 ng/ml murine bFGF (PeproTech EC, London, UK). Cells at days 4 and 8 of differentiation were collected to evaluate expression levels of differentiation markers and apoptosis-associated miRNAs. A positive control for neural differentiation was also included at day 8 using LY411575, a γ-secretase inhibitor known to inhibit the Notch pathway and thus enhance neuronal differentiation. Cells were supplemented with either 0.01% DMSO (control) or 10 nM LY411575 (in 0.01% DMSO) for 12 hours. LY411575-treated cells and respective controls were photographed under an inverted microscope Leica DMIL using a DC200 camera (Leica Wetzlar, Germany)

#### PC12 Cells

Rat adrenal pheochromocytoma (PC12) cells were propagated in RPMI 1640 medium (Invitrogen Corp.) supplemented with 10% horse serum (Invitrogen Corp.), 5% FBS (Invitrogen Corp.) and appropriate antibiotics in an humidified incubator maintained at 37°C with 5% CO_2_. PC12 cells were differentiated in RPMI 1640 medium containing 1% horse serum and antibiotics with either none, 5 or 50 ng/ml mouse nerve growth factor, (NGF 2.5S) (Chemicon, Temecula, CA) at 24 hours. Differentiation medium was replaced and fresh NGF 2.5 D was added every third day to differentiating cells. Cells were collected at 1, 2, 4 and 7 days prior to differentiation to evaluate expression levels of apoptosis-associated miRNAs.

#### NT2N Cells

The human teratocarcinoma-derived Ntera2/D1 neuron-like (NT2N) cells were obtained based on standard protocols with minor alterations [34,35]. Briefly, cells were differentiated by treating adherent dividing cells with 10 μM all-*trans *retinoic acid (Sigma Chemical Co.) for up to 3 weeks. Cells were collected at various time points (0, 14 and 21 days); and for some a replate onto Matrigel coated culture dishes after 14 days of retinoic acid was performed. Replated cells were incubated with mitosis inhibitors (1 μM cytosine D-arabinofuranoside, 10 μM fluorodeoxyuridine, 10 μM uridine) (Sigma Chemical Co.), in the absence of retinoic acid, and collected 7 days after treatment to evaluate expression levels of apoptosis-associated miRNAs.

### RNA isolation and semiquantitative reverse-transcriptase-polymerase chain reaction (RT-PCR)

Total RNA was isolated from mouse NS cells, PC12 and NT2N cells by TRIzol reagent (Invitrogen Corp.) according to manufacturer's protocol. ES cell RNAs were extracted using High Pure RNA Isolation kit (Roche Diagnostics), with the inclusion of DNAse I treatment according to manufacturer's instructions. For semiquantitative RT-PCR, 0.5-1 μg of total RNA from NS and ES cells were reverse-transcribed using oligo(dT) (Integrated DNA Technologies Inc., Coralville, IA) and SuperScript II reverse transcriptase (Invitrogen Corp.). Specific oligonucleotide primer pairs were incubated with cDNA template for PCR amplification using the Expand High Fidelity^PLUS ^PCR System from Roche Applied Science. The following sequences were used as primers: Nestin sense 5'-CTGGAACAGAGATTGGAAGGCCGCT-3'; Nestin antisense 5'-GGATCCTGTGTCTTCAGAAAGGCTGTCAC-3'; Mash1 sense 5'-AGATGAGCAAGGTGGAGACG-3'; Mash1 antisense 5'-TGGAGTAGTTGGGGGAGATG-3'; Ngn1 sense 5'-ATGCCTGCCCCTTTGGAGAC-3'; Ngn1 antisense 5'-TGCATGCGGTTGCGCTCGC-3'; Ngn2 sense 5'-ACCGCATGCACAACCTAAAC-3'; Ngn2 antisense 5'-AGCGCCCAGATGTAATTGTG-3'; b-III tubulin sense 5'-AATGAGGCCTCCTCTCACAA-3'; b-III tubulin antisense 5'-CTTGCTGATGAGCAGTGTGC-3'; GFAP sense 5'-CCAAACTGGCTGATGTCTACC-3'; and GFAP antisense 5'-GCTTCATGTGCCTCCTGTCTA-3', GAPDH sense 5'-ATTCAACGGCACAGTCAAGG-3'; and GAPDH antisense 5' TGGATGCAGGGATGATGTTC-3'. The ribosomal RNA subunit 28 S was used as control when using mouse NS cells, while GAPDH was used as control in mouse ES cells.

### Preparation of labeled RNA and array hybridization

Microarray analysis was performed using a custom microarray platform, highly sensitive, allowing the profiling of even weakly expressed miRNAs [36]. Briefly, a miRNA probe set containing ~1140 oligonucleotides as probes, complementary to *Caenorhabditis elegans, Drosophila*, zebra fish, mouse, rat and human miRNAs was purchased from Invitrogen. The set also included a number of internal and negative control probes. Oligonucleotides were printed in quadruplicates on Corning GAPSII-coated slides in the Microarray Facility at the University of Minnesota. For RNA labeling, 25 μg of total RNA was ligated to 0.5 μg of a synthetic linker, pCU-DY547 (Dharmacon, Lafayette, CO, USA). To control the hybridization process, reference DNA oligonucleotides complementary to a subset of mammalian miRNAs were combined and labeled with a ULYSIS Alexa Fluor 647 Kit (Invitrogen Corp.). Labeled RNAs and DNAs were then mixed and hybridized to microarray slides [37]. Finally, slides were scanned with a ScanArray 5000 machine (Perkin Elmer, Waltham, MA, USA). Microarray images were processed with Bluefuse software (BlueGnome, Cambridge, UK) to quantify pixel intensities. Individual spots on the slides were further inspected to exclude abnormal spots from subsequent calculations.

### Evaluation of miRNAs expression levels by quantitative Real Time-PCR

Real-time PCR was performed in an Applied Biosystems 7300 Sequence Detection System (Applied Biosystems, Foster City, CA). Ten nanograms of total RNA were reverse transcribed using a TaqMan^® ^MicroRNA Reverse Transcription (RT) kit from Applied Biosystems. Each RT reaction contained 1x stem-loop RT specific primer, 1x reaction buffer, 0.25 mM each of dNTPs, 3.33 U/μl Multiscribe RT enzyme and 0.25 U/μl RNase inhibitor. The 15-μl reactions were incubated for 30 min at 16°C, 30 min at 42°C, and 5 min at 85°C and then held at 4°C. The PCR reaction was performed using a standard TaqMan^® ^PCR kit protocol (Applied Biosystems). Briefly, following the RT step, 1.33 μl of the RT reaction were combined with 1 μl of a TaqMAn MicroRNA Assay (20x; forward primer, reverse primer and probe) and 17.67 μl of TaqMan^® ^Universal PCR Master Mix, No AmpErase^® ^UNG in a 20 μl final volume. The reactions were incubated at 95°C for 10 min, followed by 40 cycles of 95°C for 15 s and 60°C for 1 min; and were run in triplicate. miRNA expression levels relative to GAPDH (mouse NS cells, ES cells, and PC12) or RNU6B (NT2N) were calculated on the basis of ΔΔ*C*t method. GeNorm and Norm-Finder were used to identify the best stable combination of reference genes/single gene for normalization in real-time PCR assays. Calibration was set using undifferentiated mouse NS cells, mouse ES cells with 4 days of differentiation or untreated with LY411575, PC12 with 1 day of differentiation and undifferentiated NT2N cells or non-replated NT2N cells. The n-fold change in miRNAs expression was determined according to the method of 2^-ΔΔCT^.

### Immunoblotting

Steady-state levels of β-III tubulin, neuronal nuclei (NeuN) and glial fibrillary acidic protein (GFAP) were determined by Western blot. Total protein extracts of mouse NS cells were prepared in lysis buffer, following standard established protocols. Protein content was measured by the Bio-Rad protein assay kit according to the manufacture's specification, using bovine serum albumin as standard. 50-80 μg of total protein extracts were separated on 12% sodium dodecyl sulphate-polyacrylamide electrophoresis gel and then subjected to immunoblots using primary mouse monoclonal antibody reactive to β-III tubulin (Tuj1, Covance, Princeton, NJ), NeuN (MAB377, Chemicon International), and GFAP (MAB360, Chemicon International). Blots were subsequently incubated with secondary antibodies conjugated with horseradish peroxidase (Bio-Rad Laboratories, Hercules, CA, USA). Finally, membranes were processed for protein detection using Immobilon (Millipore Corporation, Billerica, MA) or SuperSignal reagent (Pierce, Rockford, IL). Ponceau S staining was used to assess equal gel loading [38].

### Immunocytochemistry

Mouse NS cells were fixed with paraformaldehyde (4% w/v) in phosphate-buffered saline (PBS) and blocked for 1 hour at room temperature in PBS containing 0.1% Triton-X-100, 1% FBS, and 10% normal donkey serum (Jackson ImmunoResearch Laboratories, Inc., West Grove, PA). Subsequently, cells were incubated with either anti-β-III tubulin (rabbit, polyclonal; Chemicon International) or anti-GFAP antibodies (mouse, monoclonal; Chemicon International) at a dilution of 1:1000 in blocking solution, overnight at 4°C. Cells were then incubated with either anti-mouse or anti-rabbit IgG conjugated to AMCA (Jackson ImmunoResearch Laboratories, Inc.) and Alexa 594 (Molecular Probes-Invitrogen), respectively, for 2 hour at room temperature. Samples were mounted using Fluoromount-G™ (Beckman Coulter, Inc.). Fluorescence microscopy assessments were performed with a Zeizz AX10 microscope (Carl Zeiss, Jena, Germany) equipped with a Leica DFC490 camera (Leica Weitzlar, Germany). Living undifferentiated cells were photographed under an inverted microscope Leica DMIL with a DC200 camera (Leica Weitzlar, Germany).

### Assessment of apoptosis

Apoptosis was assessed by evaluating DNA fragmentation, nuclear morphology and phosphatidylserine exposure.

*Evaluation of DNA fragmentation by TUNEL assay*. Apoptosis-induced DNA fragmentation was determined using the transferase mediated deoxyuridine triphosphate (dUTP)-digoxigenin nick-end labeling (TUNEL) assay. Cells were fixed with 4% (w/v) paraformaldehyde and processed using an Apoptag *in situ *apoptosis detection kit according to the manufacturer's protocol (Chemicon Int., Temecula, CA). The samples were examined using a Leica DM 2500 bright-field microscope (Leica Weitzlar, Germany).

*Morphologic evaluation of apoptosis*. Hoechst labeling of mouse NS cells was used to detect apoptotic nuclei. In brief, the medium was gently removed at the indicated times with minimal detachment of the cells. Attached cells were fixed with 4% (w/v) paraformaldehyde in PBS, pH 7.4, for 10 minutes at room temperature, incubated with Hoechst dye 33258 (Sigma Chemical Co.) at 5 μg/ml in PBS for 5 minutes, washed with PBS and mounted using PBS:glycerol (3:1, v/v). Microscopy assessments were performed with a Zeizz AX10 microscope (Carl Zeiss, Jena, Germany) equipped with a Leica DFC490 camera (Leica Weitzlar, Germany). Normal nuclei showed non-condensed chromatin dispersed over the entire nucleus. Apoptotic nuclei were identified by condensed chromatin, contiguous to the nuclear membrane, as well as nuclear fragmentation of condensed chromatin.

*Cytofluorometric analysis of apoptosis*. Apoptotic cells were quantified by cytofluorometric analysis using a FACSCalibur (Becton Dickinson, Mountain View, CA) as described previously [39]. Cells were stained with the vital dye propidium iodide (PI; 5 μg/mL; Sigma, Steinheim, Germany) and concomitantly with Annexin-V-APC (eBioscience, Inc.) according to manufacturer's instructions to determine the phosphatidylserine exposure. Data were statistically evaluated using FlowJo software (Tree Star, Inc, Ashland, OR).

### Flow Cytometry

Cells were trypsinized (0.025% trypsin/EDTA; Invitrogen Corp.) and harvested in Ca^2+^-free and Mg^2+^-free PBS and 2% FBS. After washing, 0.5-1 × 10^6 ^cells were fixed with paraformaldehyde (4% w/v) in PBS for 20 min on wet ice, and blocked for 20 min in PBS, containing 0.25% saponin (Fluka, Biochemika, Switzerland) and 5% normal donkey serum (Jackson ImmunoResearch Laboratories, Inc., West Grove, PA). Subsequently, cells were incubated with anti-NeuN (MAB377; Chemicon International) at a dilution of 1:20 in PBS, containing 0.1% saponin and 5% normal donkey serum, for 30 min. Cells were then incubated with anti-mouse antibody conjugated to Cy5 (Jackson ImmunoResearch Laboratories, Inc.), for 20 min. Cells were analyzed using a FACSCalibur (Becton Dickinson, Mountain View, CA).

### Statistical Analysis

Results from different groups were compared using the Student's t test, or one-way ANOVA. Kruskal-Wallis or the Mann-Whitney U tests were also used whenever the assumptions of the parametric test were not satisfied. Values of *p *< 0.05 were considered statistically significant. All statistical analysis was performed with GraphPad InStat software (GraphPad Software, Inc, San Diego, CA).

## Results

### NS cells have both neurogenic and gliogenic potential *in vitro*

**Figure 1:**
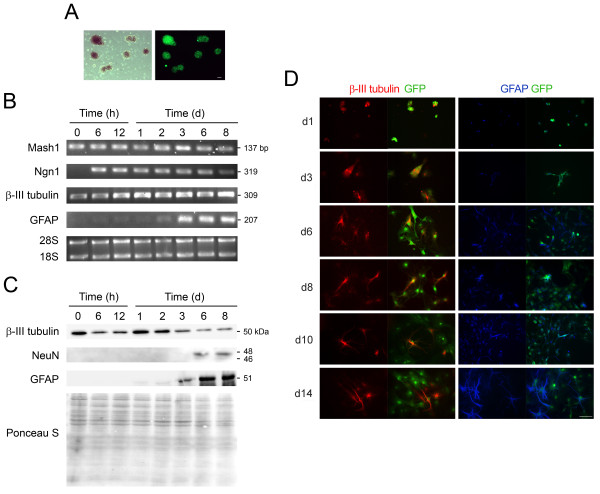
**Mouse NS cells have both neurogenic and gliogenic potential *in vitro***. Mouse NS cells were expanded in the presence of EGF and bFGF, and induced to differentiate as described in *Materials and Methods*. Cells were harvested for total RNA and protein extraction, or fixed at different time points and processed for immunostaining analysis. Neuronal differentiation was readily detected after incubation in differentiation medium, while glial production was only observed from day 3. A) Mouse NS cells constitutively expressing GFP were grown in suspension as neurospheres that increased in number and size with cell proliferation (phase contrast and GFP fluorescence images). B) Semi-quantitative RT-PCR analysis for selected markers of lineage commitment at successive time-points of mouse NS cell differentiation. Neurospheres (undifferentiated NS cells) collected before platting in differentiation medium were considered 0 time differentiation. Data was normalized to 28 S. C) Representative immunoblots showing protein expression of neuronal (β-III tubulin and NeuN) and glial (GFAP) markers during mouse NS cell differentiation. Ponceau S was used as loading control. D) Mouse NS cells labeled with anti-β-III tubulin and anti-GFAP antibodies, to visualize neurons and glial cells, respectively. Representative immunostaining images were from at least 3 independent experiments. Scale bar: 50 μm. Ngn1, neurogenin1; GFAP, glial fibrillary acidic protein; NeuN, neuronal nuclei; h, hours; d, days.

It is known that both embryonic neural tissue and certain regions of the adult vertebrate central nervous system contain resident populations of progenitor/precursor stem cells [40]. NS cells can be isolated and expanded as undifferentiated floating clusters called neurospheres in the presence of bFGF and/or EGF [41]. In addition, NS cells have the ability to differentiate into neurons, astrocytes and oligodendrocytes in a time controlled fashion [42]. Thus, NS cells represent a powerful *in vitro *model system for studying and elucidating the molecular mechanisms that underlie neural differentiation. Using mouse NS cells constitutively expressing GFP (Figure 1A), we confirmed the differentiation potential toward neuronal and glial fates by monitoring the appearance of various cell-specific markers.

The proneural basic helix-loop-helix (bHLH) transcription factor Neurogenin1 (Ngn1) was not expressed in neurospheres, while Mash1 was readily detected (Figure 1B). Following incubation in differentiation medium, cells displayed a sharp increase in markers for neurons and neural precursors. Ngn1 was readily detected at 6 hours and tended to decrease during cell differentiation. Mash1 was slightly increased but not significantly modulated. Upregulation of proneural bHLH transcription factors was associated with a subsequent increase in β-III tubulin at both the mRNA and protein levels, indicating that neurogenesis is induced early after cell incubation in differentiation medium. At the protein level, β-III tubulin expression peaked at 1 day of differentiation and gradually decreased thereafter (Figure 1C). These results suggested that the neurogenic potential of mouse NS cells rapidly decreased with differentiation.

In cell culture, there is an inverse relationship between Ngn expression and the propensity of the neural progenitors to undergo differentiation into astrocytes, suggesting that proneural bHLH factors might act to suppress the formation of astrocytes [43]. In agreement with these observations, the glial marker GFAP was only detected at day 3, in association with decreased levels of Ngn1 and concomitant with a reduction in the number of neurons. Importantly, analysis of NeuN protein expression revealed postmitotic neurons after 6 days of differentiation. Finally, analysis by immunocytochemistry using β-III tubulin and GFAP markers further confirmed the decreased neurogenic potential of mouse NS cells throughout differentiation, and the generation of glial cells after the peak of neuronal production (Figure 1D).

### Apoptosis-associate miRNAs are differentially modulated during mouse neural stem cell differentiation

miRNAs are thought to play a role in cancer pathogenesis. In fact, a specific subset of miRNAs is upregulated in most cancer profiles, and are, therefore, considered to be antiapoptotic miRNAs [44]. The best characterized of that subgroup are miR-21, miR-222, miR-221, miR-17-92 cluster, miR-106 and miR-155. In contrast, proapoptotic miRNAs are usually downregulated in cancer, and include miR-15, miR-16, the let-7 family and members of the miR-34 family. Importantly, we have recently shown that apoptosis-associated factors temporally modulate mouse NS cell differentiation [45]. As miRNAs have recently been implicated in neural differentiation [46], we sought to investigate whether apoptosis-specific miRNAs are particularly relevant during mouse NS cell differentiation.

Global miRNA expression profiling studies revealed differential expression patterns of apoptosis-related miRNAs throughout mouse NS cell differentiation (Tables 1 and 2). In general, let-7 family members were upregulated at 3 days, and decreased at 8 days of differentiation. Interestingly, and similar to miR-34a/b/c, miR-16 was upregulated at both 3 and 8 days of neural differentiation. In contrast, antiapoptotic miRNAs were in general downregulated at 8 days. Further analysis of the differentially expressed miRNAs involved clustering analysis into different groups, according to variations in the apoptosis-associated miRNAs expression (Additional file 1). Less differentiated cells (1 and 3 days) were grouped separately from those that were more differentiated (8 days). Importantly, similar grouping was not achieved in clustering analysis using all detected miRNAs.

**Figure 2:**
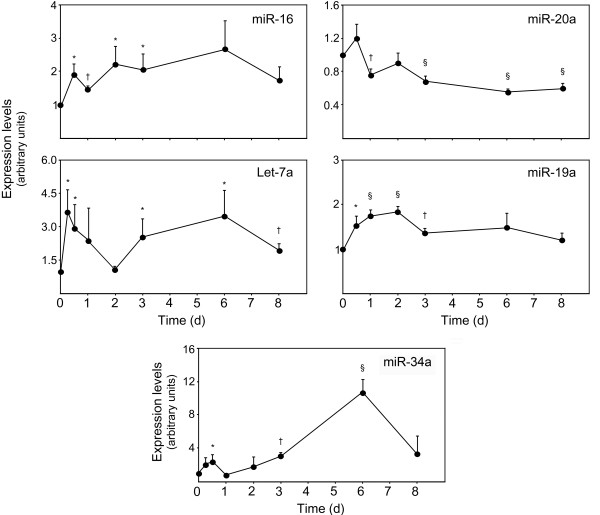
**Apoptosis-associated miRNAs are modulated during mouse NS cell differentiation**. Expression of specific proapoptotic (miR-16, let-7a and miR-34a) and antiapoptotic miRNAs (miR-20a and miR-19a) were analyzed by quantitative Real Time-PCR from 10 ng of total RNA using specific Taqman primers and GAPDH for normalization. *p < 0.05, †*p *< 0.01 and §*p *< 0.001 compared to day 0 (undifferentiated cells). Expression levels were calculated by the ΔΔ*C*t method using undifferentiated cells as calibrator. Data represent mean ± SEM of four independent experiments.

Based on a possible link between miR-16, let-7a and miR-34a with known apoptotic molecules that have already been associated with differentiation, we decided to validate microarray data for the three proapoptotic miRNAs throughout mouse NS cell differentiation by quantitative real time-PCR (Figure 2). Antiapoptotic miR-19a and miR-20a, two miR-17-92 cluster members were also investigated. Proapoptotic miRNAs were confirmed to be upregulated during mouse NS cell differentiation. Notably, miR-16 expression levels were significantly increased from 12 hours to 3 days of differentiation, when compared with undifferentiated cells (*p *< 0.05) (Figure 2). Still, its expression levels remained elevated throughout differentiation. Similarly, let-7a was significantly upregulated following induction of differentiation, increasing almost 4- and 3-fold at 6 and 12 hours, respectively (*p *< 0.05). After returning to basal levels at day 2, let-7a expression was again significantly upregulated at day 3 and 6, by 2.6- and 3.5-fold, respectively (*p *< 0.05), slightly decreasing at day 8 (*p *< 0.01). miR-34a, in turn, was modulated primarily beyond the third day of differentiation. After a 3-fold increase at 3 days (*p *< 0.01), miR-34a was further upregulated at day 6 by ~ 11-fold (*p *< 0.001), comparing with undifferentiated cells. Similarly to miR-16 and let-7a, a decrease in miR-34a expression occurred from day 6 to day 8.

The synchronous induction of proapoptotic miRNAs suggested that they may play a role during mouse NS cell differentiation. In contrast, antiapoptotic miR-20a was mainly downregulated throughout neural differentiation. In fact, its expression levels were ~ 2-fold downregulated from 6 days and beyond (*p *< 0.001), compared to undifferentiated cells. Curiously, miR-19a expression was mostly increased during the first 3 days of differentiation, gradually decreasing toward control levels. The onset of miR-19a expression correlates with induction of proliferation during the first 3 days of differentiation (data not shown).

### Expression of apoptosis-related miRNAs is not associated with increased cell death during mouse NS cell differentiation

**Figure 3:**
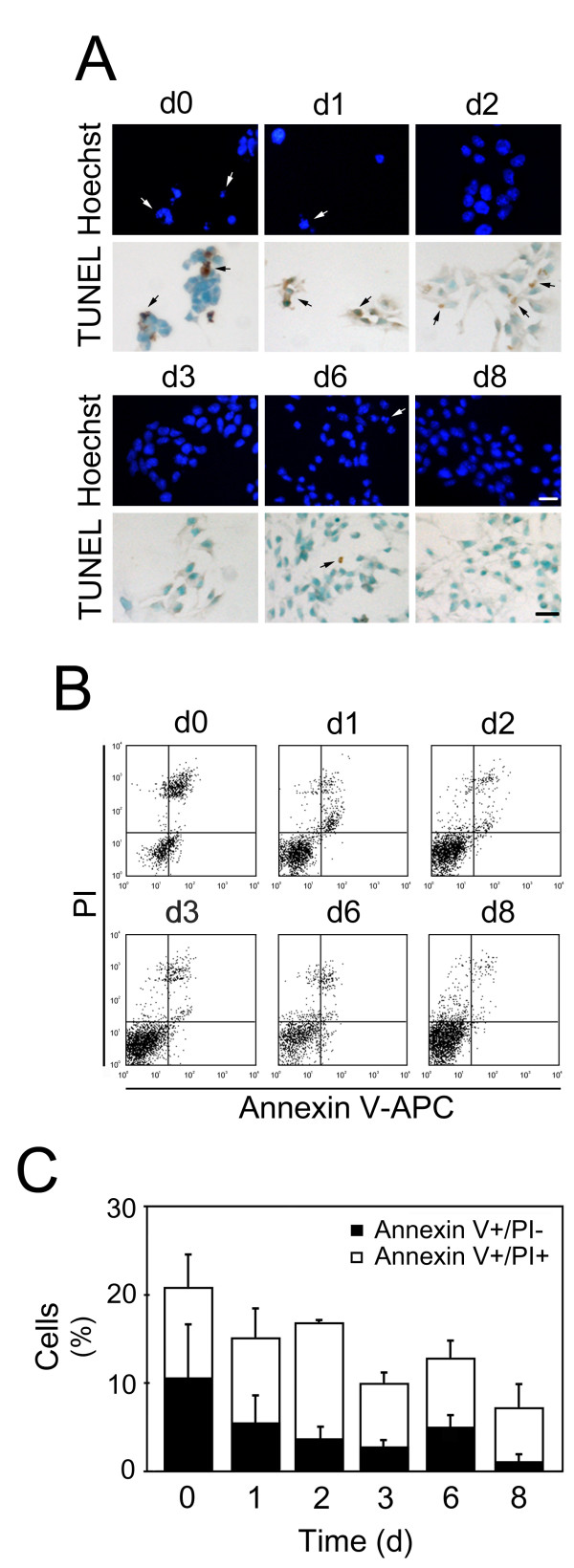
**Differentiation of mouse NS cells is not associated with increased cell death**. Mouse NS cells at different days of differentiation were fixed and processed for either morphological evaluation of apoptosis or TUNEL assay, or were stained with Annexin-V-APC/PI. A) Evaluation of apoptosis by Hoechst staining and TUNEL assay. Representative images from at least three independent experiments. Arrows indicate apoptotic cells. B) Representative FACS diagrams depicting the percentages of either dying (Annexin+/PI-) or dead (Annexin+/PI+) cells. C) Quantitation of the data depicted in FACS diagrams. Results are mean ± SEM. This experiment was repeated at least three times, yielding comparable results. Scale bar: 10 μm. d, days.

To exclude the possibility that expression of proapoptotic miRNAs is associated with increased cell death, we evaluated cellular apoptosis during mouse NS cell differentiation using Hoechst staining, TUNEL assay and cytofluorometric analysis. Undifferentiated cells exhibited major apoptosis-associated alterations, including chromatin condensation and nuclear fragmentation (Figure 3A, *Hoechst*), DNA fragmentation (Figure 3A, *TUNEL*), exposure of phosphatidylserine on the outer leaflet of the plasma membrane (detected with annexin V-APC conjugates) (Figure 3B and 3C), as well as membrane permeabilization, as indicated by the staining with the vital dye PI. Importantly, induction of cell differentiation resulted in minor alterations in nuclear morphology, while DNA fragmentation was slightly detected during the first two days of differentiation. In addition, phosphatidylserine exposure was only slightly increased from day 3 to day 6, but always less than 6% during differentiation. Furthermore, no significant loss of cell viability was observed. Taken together, our results indicated that differentiation of mouse NSC was not associated with a significant increase of cell death.

**Figure 4:**
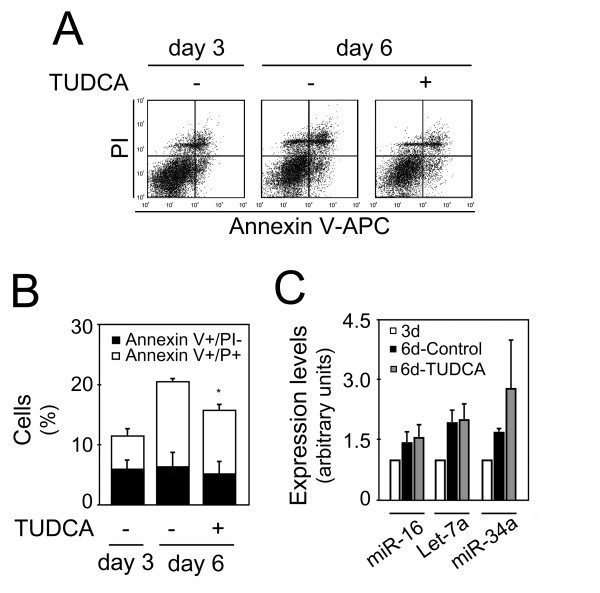
**Inhibition of apoptosis by TUDCA was not associated with a decrease in proapoptotic miRNAs expression**. Mouse NS cells with 3 days of differentiation were either untreated or treated with 50 μM of TUDCA for 72 hours. Collected cells were stained with Annexin-V-APC/PI to evaluate cell death, or processed for total RNA extraction and miRNAs expression evaluation by quantitative Real Time-PCR. A) Representative Annexin V-APC/PI stainings showing decreased cell death after TUDCA incubation. B) Quantitation of either dying (Annexin+/PI-) or dead (Annexin+/PI+) cells depicted in FACS diagrams. Results are mean ± SEM of triplicates. C) Expression of proapoptotic miRNAs at 3 and 6 days, with or without TUDCA treatment. miR-16, let-7a and miR-34a expression were evaluated from 10 ng of total RNA, using specific primers for each miRNA, and GAPDH for normalization. Expression levels were calculated by the ΔΔ*C*t method using differentiated cells at 3 days as calibrator. Data represent mean ± SEM of three independent experiments. *p < 0.05 compared to respective nontreated cells at 6 days.

All three proapoptotic miRNAs increased from day 3 to day 6 of differentiation. In addition, a concomitant increase in cell death was also observed. Thus, to exclude the role of these specific miRNAs in apoptosis, we inhibited apoptosis at 6 days and reevaluated changes on miRNA expression (Figure 4). Incubation with the antiapoptotic TUDCA for 72 hours, reduced Annexin+/PI- cells from 6.4% to 5.3% (Figure 4A and 4B). In addition, loss of cell viability (Annexin+/PI+ cells) was significantly reduced from 14.2% to 10.6% (*p *< 0.05). More importantly, the decrease in cell death after TUDCA treatment did not decrease the expression of proapoptotic miRNAs (Figure 4C). These results strongly suggested that modulation of miR-16, let-7a and miR-34a was most likely due to cell differentiation rather than cell death.

### Apoptosis-associated miRNAs are upregulated in different models of neural differentiation

**Figure 5:**
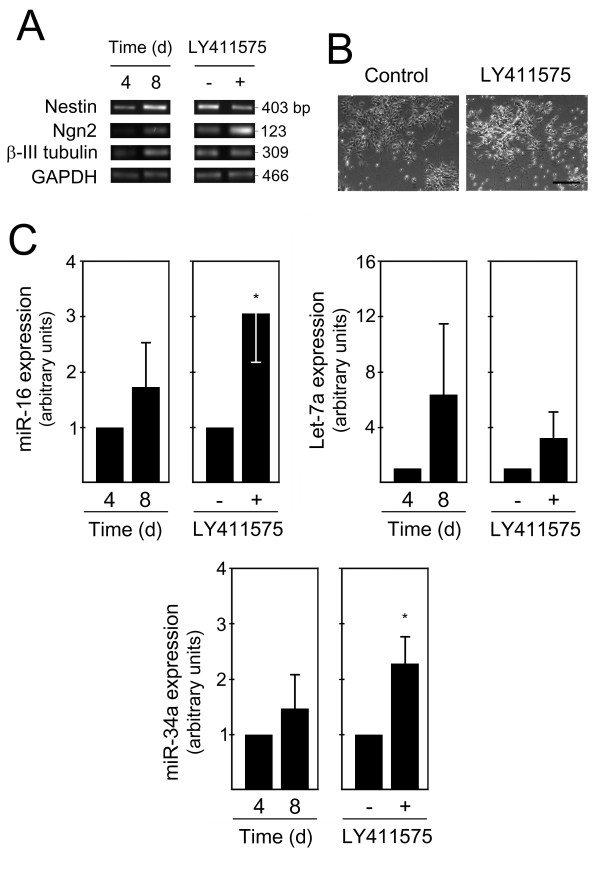
**miR-16, let-7a and miR-34a are increased during mouse ES cell differentiation**. Mouse ES cells (Sox1-GFP 46C) were differentiated using an adherent monolayer protocol. Cells at 4 and 8 days were collected for total RNA extraction and subsequently processed for evaluation of specific differentiation markers, as well as proapoptotic miRNA expression by quantitative Real Time-PCR. A positive control for neural differentiation was also performed at day 8 by treating cells with either 10 nM LY411575 or 0.01%DMSO (control) for 12 hours. A) Semi-quantitative RT-PCR analysis for selected markers of lineage commitment in day 4 and 8, as well as in LY411575-treated and untreated cells. B) Representative bright-field, phase contrast images showing increased neuronal differentiation after LY411575 incubation compared with control (DMSO-treated) cells. C) Expression of miR-16, Let-7a and miR-34a at 4 and 8 days of ES cell differentiation and in control (DMSO-treated) and LY411575-treated rosette cultures at 8 days. miRNAs expression were evaluated from 10 ng of total RNA, using specific primers for each miRNA, and GAPDH for normalization. Expression levels were calculated by the ΔΔ*C*t method using either differentiated cells at 4 days or LY411575-untreated cells as calibrator. Data represent mean ± SEM of three independent experiments. *p < 0.05 compared to respective nontreated cells. Scale bar: 50 μm. d, days.

To further validate the role of the proapoptotic miRNAs, miR-16, let-7a and miR-34a in neural cell differentiation, we investigated whether they were upregulated in other neural differentiation models, including mouse ES cells, PC12 and NT2N cell lines. The generation of neurons from ES cells has reproducibly been characterized *in vitro *[32,33]. Neural progenitors derived from ES cells organize themselves into rosette-like structures that can subsequently differentiate into neurons, astrocytes and oligodendrocytes. Using the same differentiation protocol, neurogenesis was evidenced by increased Ngn2 and β-III tubulin mRNA expression levels at 8 days of differentiation (Figure 5A). The neural progenitor marker Nestin was also increased, suggesting a concomitant increase in neural progenitors. In fact, at this stage of differentiation, expression levels of miR-16, let-7a and miR-34a were increased when compared with day 4 (Figure 5B). The inhibition of Notch pathway at 8 days of differentiation by LY411575 treatment resulted in increased neuronal production. In fact, neuronal markers, particularly Ngn2, were increased, and morphological evaluation was consistent with the presence of differentiated cells (Figure 5A and 5C). Notably, LY411575-induced neurogenesis resulted in a significant increased of miR-16 and miR-34a, by 3- and 2-fold (Figure 5B), respectively (*p *< 0.05), supporting the potential involvement of both miRNAs in neuronal differentiation. Glial differentiation was not detected in the time points analyzed (data not shown).

**Figure 6:**
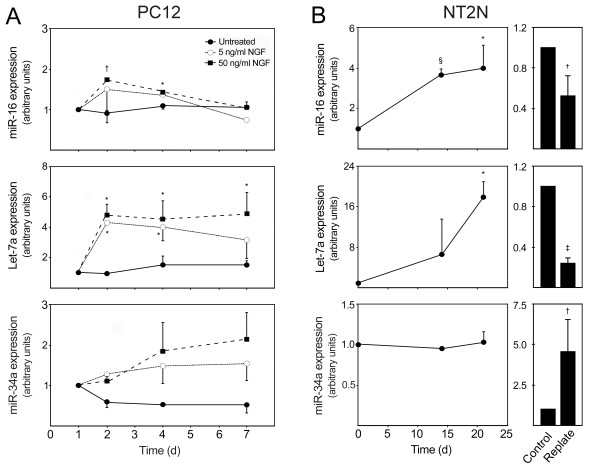
**Differentiation of PC12 and NT2N cells were associated with modulated levels of miR-16, let-7a and miR-34a expression**. PC12 cells were differentiated upon NGF treatment at 1 day with either 5 or 50 ng/ml. NGF-untreated cells were also prepared. Cells were collected at 1, 2 4 and 7 days. NT2N cells were induced to differentiate with 10 μM all-trans retinoic acid and collected at 0, 14 and 21 days. In parallel experiments, cells were replated at 14 days in Matrigel, deprived of retinoic acid and incubated with mitotic inhibitors and further cultured for 7 days. Control cells were maintained in initial retinoic acid treatment. Both PC12 and NT2N-collected cells were processed for total RNA extraction and evaluation of miRNAs expression levels using quantitative Real Time-PCR. A and B) miR-16, let-7a and miR-34a expression during PC12 and NT2N differentiation, respectively. miRNAs expression were evaluated from 10 ng of total RNA, using specific primers for each miRNA. GAPDH and RNU6B were used for normalization in PC12 and NT2N cells, respectively. Expression levels were calculated by the ΔΔ*C*t method. NGF-untreated cells at 1 day was used as calibrator in PC12 cells, while undifferentiated (day 0) or unreplated cells were used as calibrator in NT2N cells. Data represent mean ± SEM of three independent experiments. A) *p < 0.05 and ^†^*p *< 0.01 compared to 1 day. miR-16 and let-7a expressions in cells treated with 5 and 50 ng/ml NGF were also significantly different from NGF untreated cells at day 4 (*p *< 0.05) and day 2 (*p *< 0.01), respectively. B) *p < 0.05 and ^§^*p *< 0.001 compared to day 0; ^†^*p *< 0.05 and ^‡^*p *< 0.001 compared to unreplated cells. d, days.

It has been previously described that differentiation of PC12 cells can be induced by NGF [47]. Cell differentiation increases during the first 7 days of treatment with NGF, as measured by neuronal outgrowth and mRNA levels of specific neurofilaments [48]. Moreover, this correlation decreases with lower NGF concentrations. Our results demonstrated that treatment of PC12 cells with 50 ng/ml of NGF markedly induced miR-16 expression at 2 and 4 days, compared with NGF-untreated cells (Figure 6A). Indeed, miR-16 expression increased by 1.8- (*p *< 0.01) and 1.4-fold (*p *< 0.05) at 2 and 4 days, respectively. Nevertheless, no difference in miR-16 expression levels was detected at 7 days, under different NGF treatments. Interestingly, 24 hours after treatment with 50 ng/ml of NGF, let-7a expression was ~ 5-fold upregulated in differentiated PC12 cells (*p *< 0.05). Under identical conditions, let-7a expression remained elevated throughout the time course (*p *< 0.05). Notably, miR-34a expression was also gradually upregulated after NGF treatment. Although to a lesser extent, similar results were obtained for all three miRNAs under suboptimal NGF 5 ng/ml concentrations. None of the selected miRs were modulated in NGF-untreated cells.

Human teratocarcinoma NT2 cells generate postmitotic neurons (NT2N) in response to retinoic acid and mitotic inhibitors [49]. Retinoic acid treatment of NT2 cells induced neuronal differentiation as evidenced by the expression of the neuronal marker NeuroD at 14 and 21 days, as well as increased expression of β-III tubulin (data not shown). In NT2N, miR-16 expression was increased by 3.6-fold at day 14 (*p *< 0.001) and almost 4-fold at day 21 (*p *< 0.05) of differentiation (Figure 6B). Similarly, let-7a was highly upregulated, particularly at 21 days of retinoic acid treatment (*p *< 0.05). In contrast, miR-34a was not modulated during cell differentiation. Replated cells showed increased differentiation, as evidenced by the increase in β-III tubulin-positive cells and detection of NeuN-positive cells (data not shown). Surprisingly, increased differentiation after replating was associated with a significant decrease in both miR-16 and let-7a expression by ~ 2 (*p *< 0.05) and 5-fold (*p *< 0.001), while miR-34a increased by 4.5-fold (*p *< 0.05), compared with non-replated cells.

**Figure 7:**
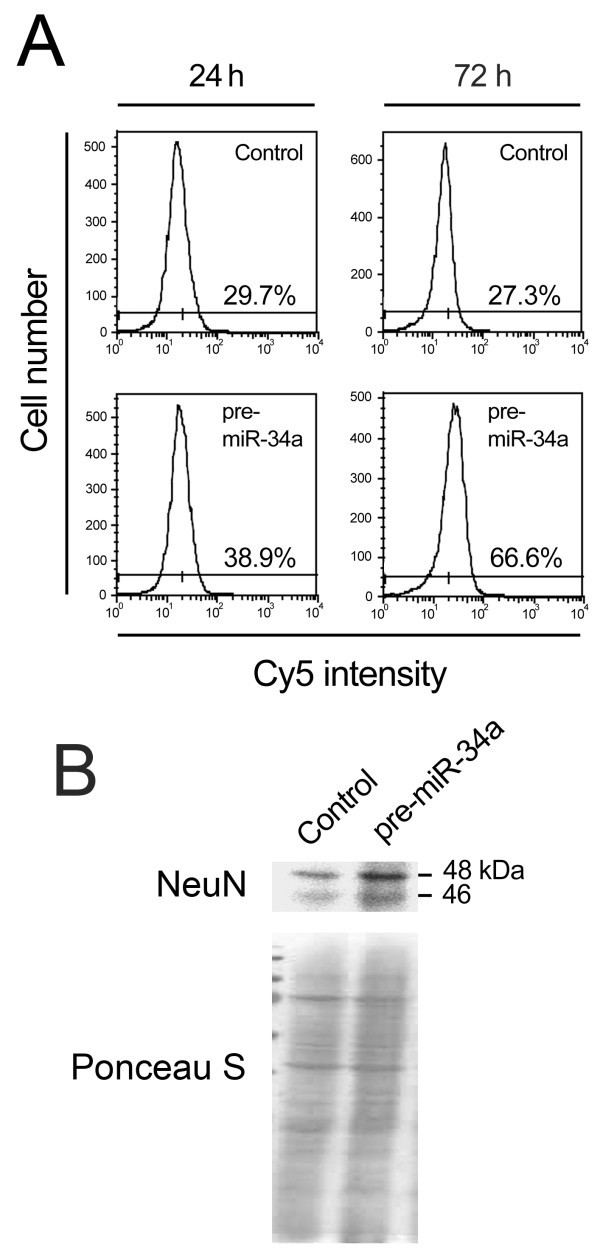
**miR-34a overexpression increases the ratio of postmitotic neurons in mouse NS cells**. Mouse NS cells were transfected using 100 nM of either control or pre-miR-34a, and collected 24 and 72 h after transfection. Collected cells were labeled for NeuN detection by flow cytometry, or processed for total protein extraction and NeuN expression evaluation by Westen blot. A) Percentage of NeuN positive cells in negative control and pre-miR-34a-transfected cells, 24 and 72 h after transfection. In independent repetitions of these experiments, similar increases in the relative number of NeuN-positive cells were observed in cultures overexpressing miR-34a when compared to control pre-miR-transfected cells. B) Representative blot showing increased NeuN expression in pre-miR-34a transfected cells.

miR-34a was the most significantly upregulated miRNA during mouse NS cell differentiation (~ 11-fold between days 3 and 6; *p *< 0.001) compared to other apoptosis-associated miRNAs. Because miR-34a upregulation coincides with the onset of postmitotic neurons, we investigated the effect of miR-34a upregulation in this population by evaluating NeuN expression. We used flow cytometry to determine the proportion of the postmitotic neuronal population (Additional file 2). The proportion of NeuN-positive cells was significantly increased when compared to cells transfected with unrelated RNA duplexes (Figure 7A). In fact, NeuN-positive cells increased from 29.7 to 38.9% 24 h after transfection. This increase was further enhanced from 27.3 to 66.6% at 72 h post-transfection. In addition, these results were confirmed by Western blot analysis (Figure 7B). No changes were detected in the proportion of astrocytes in the pre-miR-34a transfected cells (data not shown). These results indicated that miR-34a contributes to neuronal differentiation in mouse NS cells.

## Discussion

Differentiation of NS and ES cells *in vitro *has attracted wide interest as an experimental system for investigating specific molecular pathways of cell development. Here, we have demonstrated neuronal and glial conversion of mouse NS cells. In this model, the neuronal population is formed mainly during the first 2 days, as indicated by the upregulation of β-III tubulin. Nevertheless, commitment to mature neurons was confirmed by the expression of NeuN at 6 days, which indicates the presence of immature postmitotic neurons. Astrocytic population peaked at 6 days, and this was maintained throughout differentiation.

Functional studies of apoptosis-associated miRNAs have focused intensely on cancer research. However, our results demonstrate that specific miRNAs regulating pro-and antiapoptotic genes may also participate in differentiation processes. The involvement of miR-16 in cell differentiation has not been previously reported. Indeed, miR-16 expression was markedly increased throughout mouse, rat and human neural differentiation. Nevertheless, the onset of miR-16 expression during mouse NS cell differentiation was not associated with the appearance of any specific cell type. miR-16 was shown to be implicated in cell cycle regulation, as well as apoptosis induction by targeting Bcl-2 [25-28]. Apoptosis is not markedly regulated during mouse NS cell differentiation, and Bcl-2 expression is increased (data not shown). Therefore, a possible role for miR-16 in the context of differentiation may be associated with cell cycle control in downregulating the proliferation potential of differentiating cells. During differentiation of neural precursors, a tightly coordinated regulation of cell cycle exit is crucial for the generation of appropriate number of neurons and proper wiring of neuronal circuits [50]. Nevertheless, here-to-fore unrecognized miR-16 targets may exert distinct, yet crucial functions during cell differentiation.

The let-7 family consists of eleven very closely related genes [51]. They are highly conserved in animals from worms to humans, and their expression increases after differentiation and maturation of tissues [52]. In addition to regulating apoptosis by targeting caspase-3 [10], it was also demonstrated that let-7 family members regulate RAS and HMGA2 oncogene through the 3'UTR [53-55]. In the present study, let-7a expression was shown to be cyclic during mouse NS cells differentiation, and corresponded to the onset of neurogenesis and gliogenesis. This suggests that let-7a expression is associated with a general mechanism of differentiation rather than differentiation of specific cell types. Our results are supported by the previous report showing that let-7a is a critical regulator of neuronal differentiation [12,13]. In fact, increased let-7a expression during ES cells, PC12 and NT2N differentiation also underscores the important role of let-7a during general differentiation. Additional studies are warranted to evaluate the mechanism(s) by which let-7a regulates cell differentiation. Importantly, although highly modulated during cell differentiation, both let-7a and miR-16 were significantly expressed in neurospheres (data not shown). Therefore, it is possible that additional mechanisms exist to antagonize let-7a and miR-16 expression during NS cell differentiation.

A specific role for miR-34a during neuronal differentiation has not been reported. Interestingly, herein we showed a significant upregulation of miR-34a during mouse NS cell differentiation, and this paralleled the appearance of postmitotic neurons. In addition, *in vitro *transient overexpression of miR-34a increased the proportion of NeuN-positive cells. Given that miR-34a has been shown to regulate genes involved in cell cycle arrest, it is possible that miR-34a upregulation is related to cell cycle exit and the subsequent appearance of immature postmitotic neurons. In fact, miR-34a upregulation in ES cells after LY treatment, as well as in NT2N cells after incubation with the mitosis inhibitor, supports this hypothesis. In addition, it has been previously shown that miR-34a can suppress cell-cycle genes and induce neural phenotype in neuroblastoma tumors [56]. Importantly, it has been previously reported that miR-34a overexpression had no effect on cell cycle arrest and survival/apoptosis of astrocytes [57,58]. However, additional studies are required to confirm the influence of miR-34a in neurogenesis and evaluate whether it mediates p53 effects on cell differentiation. In fact, several potential miR-34a targets might be involved in the transition towards postmitotic neurons. It was previously demonstrated that miR-34a targets Sirt1 and is regulated by p53, which in turn, is activated by Sirt1 suppression [59]. In addition, miR-34a may also regulate the differentiation process by influencing Notch signaling pathway [57,60]. In contrast to let-7a and miR-16, miR-34a was barely detected in undifferentiated cells, supporting its specific involvement in cell differentiation.

miR-19a and miR-20a are members of the miR-17-92 cluster [61], which consists of seven mature miRNAs, previously linked to tumorigenesis. Recently, additional functions have been assigned to this cluster, particularly to miR-20a and miR-19a. Specifically, miR-20a was shown to control monocyte differentiation [62]. In fact, transfection of hematopoietic progenitors with miR-20a increased the proliferation of monocytes and blocked differentiation, whereas inhibition of miR-20a caused a decrease in proliferation and more rapid differentiation and maturation. Our results, showing that miR-20a decreases during mouse NS cell differentiation, are in agreement with this previous report.

Only limited information is available regarding the physiological role of miR-19a. It has been demonstrated that it acts as a positive regulator of antiapoptotic Stat3/IL-6 receptor signaling by directly suppressing SOCS-1, thereby facilitating malignant growth of multiple myeloma [63]. Further, miR-19 appears to affect the level of proapoptotic protein Bim, thereby preventing apoptosis and promoting cell survival. The possible role of miR-19a on cell survival, may explain why this miRNA was upregulated at early stages after the induction of mouse NS cell differentiation. However, additional studies are required to determine the specific role of both miR-20a and miR-19a during cell differentiation, and also evaluate if their expression is restricted to a specific cell type.

## Conclusions

In conclusion, our results demonstrate that apoptosis-associated miRNAs are differentially expressed during neural differentiation, in the absence of cell death, and identify miR-16, let-7a and miR-34a as important players.

## Abbreviations

NS cells: neural stem cells; ES cells: embryonic stem cells; GFP: green fluorescent protein; miR or miRNA: microRNA.

## Authors' contributions

MMA carried out mouse NS cell culture studies, RNA isolation, and real time PCR assays and drafted the manuscript. DMS performed mouse ES cell cultures, respective characterization, and RNA isolation. JMX was involved in mouse NS cell characterization. SS and CMPR were involved in experimental design and interpretation of the data. WCL, CJS, SS and CMPR contributed to the manuscript. All authors read and approved the final manuscript.

## Supplementary Material

Additional file 1 **Clustering analysis**. Clustering analysis of differentiated mouse NS cells based on apoptosis-associated miRNA expression levels at 1, 3 and 8 days. Click here for file

Additional file 2 **Flow cytometry analysis**. Forward scatter/side scatter (FSC/SSC) distribution of mouse NS cells. Unstained controls, in which incubation with primary antibody was avoided, were performed to determine the limit at which fluorescence intensity can be considered specific. Click here for file
